# Employing core regulatory circuits to define cell identity

**DOI:** 10.15252/embj.2020106785

**Published:** 2021-05-02

**Authors:** Nathalia Almeida, Matthew W H Chung, Elena M Drudi, Elise N Engquist, Eva Hamrud, Abigail Isaacson, Victoria S K Tsang, Fiona M Watt, Francesca M Spagnoli

**Affiliations:** ^1^ Centre for Stem Cells and Regenerative Medicine Guy’s Hospital King’s College London London UK

**Keywords:** cell identity, core regulatory circuit, GRN, regenerative medicine, transcription factor, Chromatin, Transcription & Genomics, Development, Stem Cells & Regenerative Medicine

## Abstract

The interplay between extrinsic signaling and downstream gene networks controls the establishment of cell identity during development and its maintenance in adult life. Advances in next‐generation sequencing and single‐cell technologies have revealed additional layers of complexity in cell identity. Here, we review our current understanding of transcription factor (TF) networks as key determinants of cell identity. We discuss the concept of the core regulatory circuit as a set of TFs and interacting factors that together define the gene expression profile of the cell. We propose the core regulatory circuit as a comprehensive conceptual framework for defining cellular identity and discuss its connections to cell function in different contexts.

## Graphical abstract

This review summarises the regulatory layers that establish and maintain cellular identity in a dynamic environment, and surveys the methodologies required to explore these.

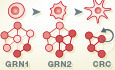

## Introduction

The nature of cell identity is a central problem in biology. Accurate identification of cell types deserves significant attention due to its impact on many areas of research and clinical applications, including regenerative medicine. Cell identities are influenced by external stimuli, such as signaling molecules, growth factors, and intercellular communication, which in turn affect downstream gene expression and jointly dictate cell phenotype and function(s) (Holmberg & Perlmann, 2012; Wagner *et al,* 2016). Even though these distinct facets of a cell's identity are interdependent, they are often considered separately. Nevertheless, the cell’s phenotype and functional characteristics ultimately represent the readout of a specific gene‐expression program. Typically, a small number of transcription factors (TF), which show a lineage‐restricted expression pattern, are considered sufficient to establish gene expression programs that define the identity of a cell (Holmberg & Perlmann, 2012; Zaret & Mango, 2016). Often, these TFs have the ability to bind to inaccessible nucleosomal DNA, acting as “pioneer” TFs (Zaret & Carroll, 2011; Zaret & Mango, 2016).

The concept that differentiated cell identity is established and continuously maintained by a set of TFs was proposed several decades ago (Blau & Baltimore, 1991). This was supported by pioneering studies with cell hybrids and heterokaryons, in which terminally differentiated cells could be successfully reprogrammed into muscle cells by cell fusion (Weiss & Green, 1967; Blau *et al,* 1983; Pomerantz *et al,* 2009), and later by gain‐of‐function approaches based on key TFs (Davis *et al,* 1987). While these experiments established that cell identity is actively maintained by TFs, it was only in 2008 that Hobert proposed the term of terminal selector gene (TSG) (Hobert, 2008). A TSG was defined as a gene that specifies individual identities by directly controlling the expression of a set of downstream differentiation genes (a.k.a. effector genes) via common cis‐regulatory motifs (a.k.a. terminal selector motifs) (Hobert, 2008). Though initially described within the context of neuron‐specific lineage determination and maintenance in *C. elegans* (Etchberger *et al,* 2007), the existence of TSGs has been confirmed in a plethora of other cell types and also in vertebrate model systems (Hobert, 2008) (Box 1). Features of neuronal cell TSG expression that may well apply to other cell types are as follows: (i) the initiation and maintenance of TSG expression are independent events; (ii) the initiation may be the result of transient expression of distinct regulatory factors, either extrinsic signals or TFs; (iii) after initiation, TSGs autoregulate their expression, ensuring continuous expression and regulation of downstream targets (Hobert, 2008, 2011).

Box 1 **Building the CRC of dopaminergic neurons**

Efforts to classify neuronal identity have greatly contributed to our understanding of CRCs, with studies in *C. elegans* being the first to conceptualize various components of CRCs such as TSGs (Hobert, 2008). For example, PBX/CEH‐20, part of the PBX TALE (three‐amino‐acid loop extension) homeodomain proteins (Selleri *et al,* 2019), was first identified to initiate and maintain the terminally differentiated state of dopaminergic (DA) neurons, thereby acting as a TSG (Doitsidou *et al,* 2013). It was later found that PBX factors, in particular Pbx1, have a conserved role in mouse midbrain DA neurons (Villaescusa *et al,* 2016). More recently, a genetic approach was used to specifically ablate *Pbx1* expression in mouse DA neurons to achieve temporal control over its expression, confirming the involvement of Pbx1 in an evolutionarily conserved CRC (Remesal *et al,* 2020). This study not only confirmed the involvement of Pbx1 in the production of dopamine, but also showed that this TF is required for the expression of a broad range of olfactory bulb DA effector genes (Remesal *et al,* 2020). Such a genetic approach enabled the distinction between the late roles of Pbx1 in terminal differentiation and preservation of neuronal identity (Remesal *et al,* 2020) and its early activities in neuroblasts as well as in midbrain DA neuron specification (Grebbin *et al,* 2016; Villaescusa *et al,* 2016) (Box 1 Figure).

**Figure:**
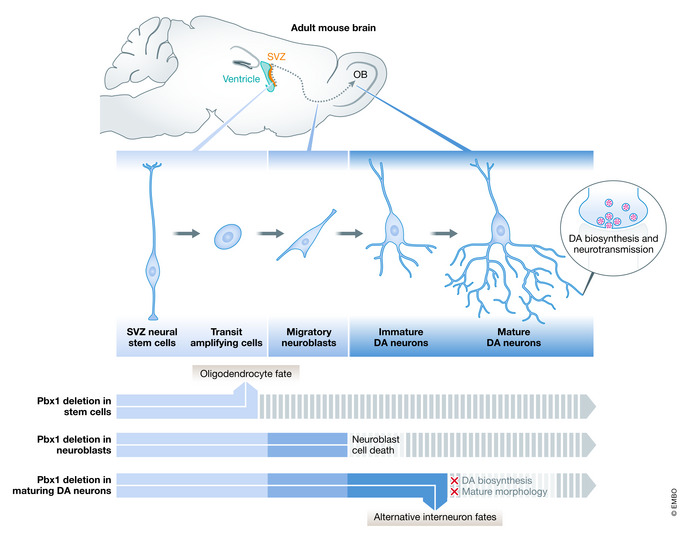
Box 1 Figure. The role of Pbx1 in the CRC of olfactory bulb DA neurons.

Pbx1 is a TF that is continuously expressed from progenitor to mature neurons. Conditional knockout approaches were key for elucidating the role of Pbx1 not only in the specification of midbrain DA neurons (Villaescusa *et al,* 2016), but also specifically in the CRC of olfactory bulb DA neurons (Remesal *et al,* 2020). DA: dopaminergic.

The transcriptional characterization of cell populations can be facilitated by the prior knowledge of TFs that promote cell identity and unfold a CRC network. For instance, the use of known DA lineage marker genes enabled Fernandes and colleagues to describe a previously unknown heterogeneity of DA neurons derived from human induced pluripotent stem cells (Fernandes *et al,* 2020). Using scRNA‐seq to obtain an unsupervised clustering of the population of cells, the group identified six distinct cell types, two being neuron progenitor populations and four being subpopulations of DA neurons. Although these populations differed in expression of certain genes, all expressed typical DA lineage markers, including Pbx1. Additionally, the *in vitro* transcriptional data overlapped well with single‐cell transcriptomic datasets of post‐mortem substantia nigra, which validated the transcriptional heterogeneity found in subpopulations of human DA neurons.

Given that DA neurons are degenerated in individuals with Parkinson’s disease, building the CRC network in DA neurons will not only enrich our understanding of this cell type, but also, potentially, contribute to the development of disease therapies (see section “Assigning functional relevance to CRCs”).

**Figure 1:**
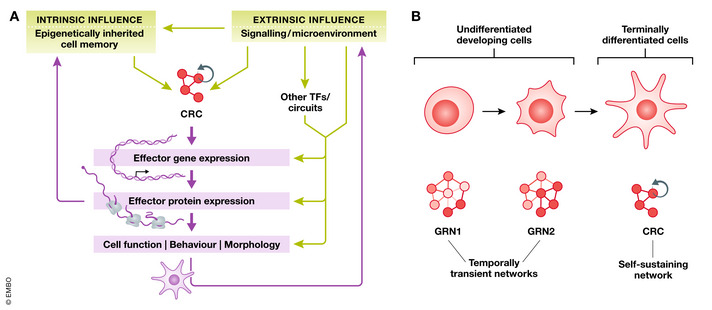
Cell identity is regulated by CRCs (A) Conceptualizing different types of information (e.g., transcriptomics, epigenomics) in the flow of biological information from DNA to function in order to shape our knowledge of CRCs. Downstream processes (purple), such as gene and protein expression, are routinely measured using transcriptomics and proteomics. Further downstream of this is cellular phenotype, a more complex readout which is measured using various assays and microscopy techniques. Factors that influence the CRC of a cell (green) include intrinsic factors and extrinsic factors, such as epigenetic memory and the external environment, respectively. While an overall flow of information is unidirectional (from top to bottom), many factors influence each other in more complex ways. (B) Model of cell identity being regulated by GRNs through development and CRCs in differentiated cells. We propose that CRCs define differentiated cell types and GRNs are temporally transient networks which drive cellular differentiation during development. GRNs adapt in response to external signals and other influences during development, resulting in a series of different developing cell states. Once cells become terminally differentiated, the TF network becomes more stable and can be defined as a CRC, which is autoregulating and activates the expression of the terminal effector gene battery. While GRNs and CRCs can be identified using similar methods, studies of GRNs additionally benefit from lineage tracing and pseudotime analysis to account for their temporal aspect.

In higher vertebrate species, acquisition of a differentiated cell identity seems to require more complex circuitries, whereby a larger panel of TFs act in a combinatorial manner (Fig 1A) (Holmberg & Perlmann, 2012). Target/effector genes are not all controlled by a similar cis‐regulatory logic, but instead different combinations of lineage‐specific TFs co‐regulate different subsets of target genes in distinct ways. Thus, only when the complete set of TFs is co‐expressed in a cell, the full repertoire of differentiation genes is induced and maintained (Holmberg & Perlmann, 2012). Davidson pioneered the concept of gene regulatory networks (GRN) governing the development of body plan and organ formation in the embryo (Davidson & Erwin, 2006). TFs and transcriptional regulators are GRN components, and their target sites are the cis‐regulatory DNA modules. Because each module is regulated by multiple TFs and each TF interacts with multiple modules, it is possible to represent developmental patterns of gene expression as an interlocking network (Peter & Davidson, 2016). Beyond early embryonic processes, GRN circuit design has been applied to describe the transcriptional control of binary fate choices in stem cell differentiation, for example, in the hematopoietic lineage (Graf & Enver, 2009; Davidson, 2010; Xia & Yanai, 2019). Furthermore, seminal studies from embryonic stem cells (ESCs) have revealed that a small set of TFs, such as NANOG, SOX2, and OCT4, called core TFs, not only bind to their own loci, but also mutually regulate one another, thereby forming cross‐regulated feed‐forward loops that maintain pluripotency (Boyer *et al,* 2005). The core TFs and their interconnected auto‐regulatory loops have been termed “core regulatory circuitry” (CRC) (Boyer *et al,* 2005; Young, 2011).

Arendt *et al* (2016) further extended these models and introduced the concept of core regulatory complex (CoRC), whereby cell type‐specific gene expression not only requires the activity of a specific combination of terminal selectors but also depends on their physical cooperativity. Based on such a model, the origin of a new cell type in evolution coincides with the occurrence of a unique CoRC, distinct from its evolutionary sister cell type (Arendt, 2008; Arendt *et al,* 2016). While the primary function of CRC or CoRC factors is to keep cells in a stable differentiated state, the notion of GRN describes a temporally hierarchical framework of gene expression that controls a differentiation process and adapts in response to external signals and other influences during development (Davidson, 2010; Marioni & Arendt, 2017) (Fig 1B).

Since CRC factors provide the ultimate instructive “code” underlying the expression of the effector genes in differentiated cells, cellular identifiers, such as the functional output, cannot be considered separately. Thus, the CRC concept might provide a standardized and comprehensive definition of a cell type, as the TF regulatory network, which is necessary for the induction and maintenance of cell type‐specific gene expression program in differentiated cells.

In this review, we discuss how CRC TFs can be employed to define cell identity in the context of differentiation strategies, which can benefit regenerative medicine.

## Identifying a core regulatory circuit

Several efforts have been made to identify individual components of cell type‐specific CRCs (Graf & Enver, 2009; Xia & Yanai, 2019). To date, most of our knowledge is based on the use of expression profiles of core TFs as a proxy for CRCs. However, to build the network, transcriptomic data need to be integrated with chromatin analyses in computational models for protein–protein, gene–protein, and regulatory element interactions (Fig 1A).

### Transcriptomics: from population to single‐cell analyses

**Figure 2:**
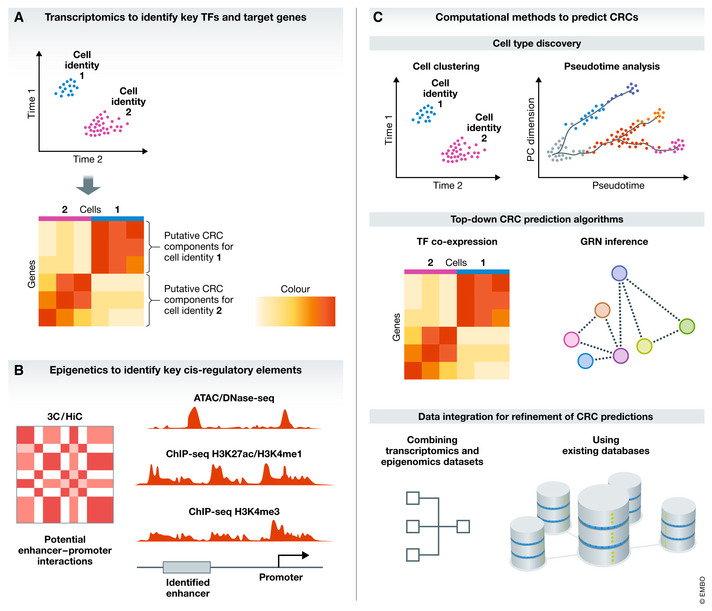
Methods employed to identify CRCs (A) Single‐cell transcriptomics allows the identification of cell populations or states (top). Putative CRC components for these cell identities can be identified by defining the TFs and downstream genes enriched in these cells (bottom). (B) Epigenetic methods allow the identification of cis‐regulatory elements that make up the CRC. Chromosome conformation capture (3C/HiC) identifies regions of DNA, which are in close contact with each other, potentially including enhancer–promoter interactions (left). ATAC‐/DNase‐ and ChIP‐seq for histone modifications identify regions of open chromatin, which can be used to identify enhancers as well as promoters and actively transcribed genes (right). (C) Computational methods are used in multiple aspects of CRC identification. Clustering of single‐cell transcriptomics data allows discovery of previously unknown cell types, while pseudotime analysis help identify transcriptional states when cell fate decisions along developmental trajectories are made (top). Several algorithms can make data‐driven predictions of CRCs by analyzing TF co‐expression and performing GRN inference (middle). Other relevant data supplied by users or deposited in databases can inform on CRC mechanisms (e.g., chromatin accessibility, promoter and enhancer states, TF‐binding and protein–protein interactions) and be integrated to refine CRC predictions (bottom).

Cell type‐enriched sets of TFs, the main components of CRCs, are still primarily discovered by transcriptome analyses (Xia & Yanai, 2019) (Fig 2A). Over the last decades, the shift from bulk transcriptomics to single‐cell or single‐nucleus RNA‐sequencing (scRNA‐seq and snRNA‐seq, respectively) has started to provide new insights into gene modules underlying individual cell types (Menon, 2018). Moreover, these approaches in genomics and transcriptomics at a single‐cell resolution have led to depositories such as the Human Cell Atlas (HCA). The HCA is a global initiative, which aims to create a comprehensive reference map of all human cell types based on their molecular profiles and their classical cellular descriptions (Regev *et al,* 2017). The purpose is to provide a unique identification of each cell type and a common framework for understanding biological processes in health and disease. Single‐cell atlases are already available for adult human tissues, including the lung, kidney, pancreas and liver, and sequencing of fetal tissues is also ongoing (https://data.humancellatlas.org/).

To date, transcriptome analyses have enabled the classification of specific mammalian brain cells in spatiotemporal and cell‐type databases (Keil *et al,* 2018; Arlotta & Paşca, 2019). The spatial transcriptome atlas of the adult human brain from the Allen Human Brain Atlas (AHBA), for example, comprises histological analysis and comprehensive microarray profiling of nearly 900 neuroanatomically precise microdissected sites of the brain in two individuals (Hawrylycz *et al,* 2012). More recently, in 2014, the U.S. National Institutes of Health funded the BRAIN Initiative Cell Census Consortium (BICCC) (Ecker *et al,* 2017). The initiative combines ten pilot projects spanning multiple approaches, including single‐cell omics and species (mice, rats, zebrafish, and humans) with the final goal to classify brain cell types based on integrated analysis of their molecular, anatomical, and physiological properties. The BICCC network works closely with the HCA to develop a comprehensive atlas of all cell types in the human body within a common coordinate framework (Regev *et al,* 2017). BICCC groups developed new technologies for profiling single neurons that identified new cell types or cell states in the nervous system (Tasic *et al,* 2016).

The availability of single‐cell data has allowed the characterization of heterogeneous transcriptional profiles, context‐dependent regulatory relationships, and functional interactomes with higher granularity (Aibar *et al,* 2017; Mohammadi *et al,* 2019). Kelley *et al* (2018) used scRNA‐seq data to examine cell‐type variations across brain regions in intact human tissue. This resulted in a robust strategy to define gene modules enriched in major neuronal subtypes, which they termed “core transcriptional identities” (Kelley *et al,* 2018; Menon, 2018).

Despite its many advantages, scRNA‐seq techniques are susceptible to several influences, which can bias the results (Chen & Zhou, 2017; Keil *et al,* 2018). Several technical factors can introduce variations in the sequencing data; cell dissociation and suspension preparation may introduce technical noise; and stress to the cell‐type viability could lead to alterations in the gene expression profiles (Ecker *et al,* 2017; Menon, 2018; Kelley *et al,* 2018). As some classes of cells are more fragile and prone to rupture than others, this will introduce bias in the populations captured. Other challenges include transcripts of short length or of low abundance in a single cell. The low amount of material may result in uneven RNA loss leading to gene drop‐out events which can be difficult to measure accurately (Chen & Zhou, 2017; Keil *et al,* 2018). In particular, Mawla and Huising have illustrated the limitations of pancreatic islets transcriptomics where the impact of endocrine cells, other than the insulin‐producing β‐cells, or auxiliary cells in the disruption of blood glucose homeostasis is often overlooked due to their lower abundance (Mawla & Huising, 2019). Although whole islet analysis is limited by the mixture of cells, which differ in abundance, Bramswig and Kaestner discussed the reliability of adding a sorting strategy to determine cell type‐specific changes (Bramswig & Kaestner, 2014).

Another challenge of developing a comprehensive human cell atlas is that scRNA‐seq requires fresh tissue and therefore relies on limited tissue donations collected either surgically or post‐mortem (Ecker *et al,* 2017; Kelley *et al,* 2018). A valuable alternative is snRNA‐seq which can be applied to archived frozen samples and provides less biased cellular coverage (Bakken *et al,* 2018). In fact, Lake *et al* (2016) revealed 16 neuronal subtypes using nuclear RNA from single nuclei harvested from post‐mortem tissue, demonstrating snRNA‐seq as a promising method to analyze the human brain. Similarly, snRNA‐seq overcame the technical problems due to rapid enzymatic RNA degradation upon resection of pancreatic tissue, which historically have led to underrepresentation of exocrine cells and hampered comprehensive sequencing of human exocrine pancreatic cells (Tosti *et al,* 2021).

An additional challenge in single‐cell transcriptomics is to classify cell variability, to define cell “types” and to distinguish them from transient cell “states”. A consensus on whether a cell going through different states should still be considered the same cell type has not yet been achieved. Xia and Yanai proposed a “periodic table” approach to distinguish cell types from cell states. Typically, scRNA‐seq analysis relies on unsupervised clustering algorithms based on the differential expression of genes to identify the cell types (Xia & Yanai, 2019). This uncovers modules of genes and provides an initial map of the relative proportions of different cell types (Regev *et al,* 2017; Menon, 2018). However, clustering based on differential gene expression might overlook the fact that cell states, such as the cell cycle or stress, are also captured (Kiselev *et al,* 2019). By contrast, by defining cell identity using the concept of CRCs, a given cell is expected to show a unique set of TFs regardless of its state, which would help to distinguish between cell types and cell states. Hence, Xia & Yanai propose a cell clustering approach that combines both differentially expressed genes and the expression profile of TFs (Xia & Yanai, 2019). This represents a practical approach for distinguishing cell states within the cluster of a given identity.

### Epigenetic modifications and chromatin landscapes

Defining CRC factors and building a network requires elucidation of the relationships between the regulators of gene expression (TFs) and the target genes (effector genes). TFs activate or inhibit the expression of genes by binding specific regulatory sequences, including promoters and enhancers (Spitz & Furlong, 2012). Identifying the enhancers that regulate genes of interest or are bound by key TFs is therefore crucial to understand the connections between the players in the CRC. As enhancers cannot be uniquely characterized by a particular sequence or feature (Coppola *et al,* 2016), they are identified using multiple approaches combined (Fig 2B).

Coordinated experiments interrogating transcriptional responses and chromatin binding via chromatin immuno‐precipitation with next‐generation sequencing (ChIP‐seq) can offer insights into different levels of gene regulation, TF‐binding motifs, DNA and chromatin modifications, and how each component is coupled to a functional output (Holmberg & Perlmann, 2012; Wilson & Filipp, 2018). Examples of CRCs in specific lineages are included in Box 1 and Box 2.

Box 2 **A CRC view of the pancreas**

Mist1 and Ptf1a, two TFs involved in the CRC of pancreatic acini, exemplify the way in which various technologies complement one another to inform our knowledge of CRCs. The function of Mist1 and Ptf1a in acinar tissue has been established thanks to mouse genetic studies (Krapp *et al,* 1996; Lemercier *et al,* 1997; Pin *et al,* 2001). Together, Mist1 and Ptf1a bind and drive the transcription of over a hundred downstream acinar genes through reiterated feed‐forward regulatory loops (Jiang *et al,* 2016). However, the depth and nature of these TFs’ involvement in acinar cell identity was not understood until more recently when a combination of epigenetic and transcriptomic analyses revealed that they are part of a CRC (Jiang *et al,* 2016). ChIP‐seq analysis revealed that Mist1 and Ptf1a share many target genes with highly juxtaposed binding sites. Ptf1a drives expression of Mist1 through binding to its enhancer, thus generating a self‐sustaining regulatory loop between the two factors capable of maintaining not only itself, but also expression of effector genes essential for acinar cell identity.

Within the endocrine compartment of the pancreas, loss‐of‐function experiments also uncovered the roles of potential CRC constituents [comprehensively reviewed in (Romer & Sussel, 2015)]. Specifically, the development of insulin‐producing β‐cells depends on several TFs such as Pdx1, Ngn3, and Nkx6.1 (Murtaugh, 2007; Best *et al,* 2008; Arda *et al,* 2013; Romer & Sussel, 2015; Jennings *et al,* 2015). While some of these developmentally crucial TFs are also members of the CRC governing terminal β‐cell identity, additional TFs such as MafA and MafB are required to maintain the mature β‐cell phenotype through regulation of downstream effector genes involved in β‐cell function (Kataoka *et al,* 2002; Matsuoka *et al,* 2004; Nishimura *et al,* 2015; Zhu *et al,* 2017; Russell *et al,* 2020).

scRNA‐seq studies have unveiled a remarkable heterogeneity within mouse and human β‐cells (Baron *et al,* 2016; Muraro *et al,* 2016; Segerstolpe *et al,* 2016; Xin *et al,* 2016; Lawlor *et al,* 2017; Mawla & Huising, 2019), which has further contributed to our understanding of these cell types. Wang and colleagues have taken advantage of single‐cell transcriptomic data to model the relationship between eight master TFs (Pdx1, Ptf1a, Nkx6.1, Sox9, Hes1, Arx, Ngn3, and Pax4) in the pancreatic cell lineage (Wang *et al,* 2020). An adaptive landscape was constructed in which states were annotated either as mature or progenitor cell types based on prior knowledge of the relationships between these factors (Wang *et al,* 2020). The model infers additional transition states along different pancreatic lineage trajectories as well as previously unrecognized progenitors characterized by distinct CRC systems (Wang *et al,* 2020).

**Figure:**
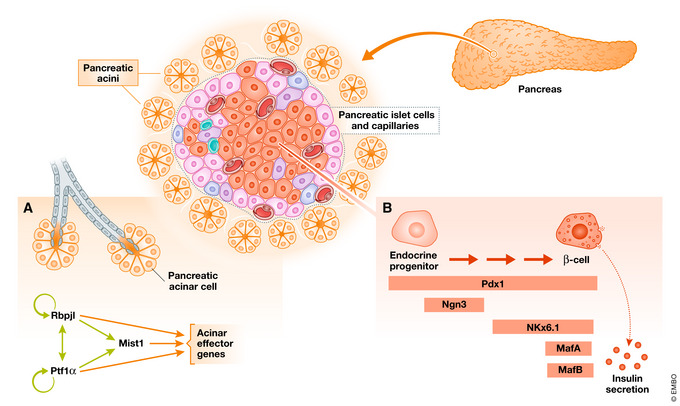
Box 2 Figure. CRCs maintain distinct endocrine and exocrine cell type in the pancreas.

The pancreas contains several highly specialized cell types with distinct physiological secretory roles; these unique cell identities are maintained by independent CRCs. (A) In the acinar CRC, Rbpjl and Ptf1a drive expression not only of acinar terminal selector genes (orange arrows), but also of themselves and other CRC members (light green arrows). This is an example of the self‐sustaining nature of CRCs. (B) Numerous TFs guide the development and maturation of the insulin‐secreting β‐cells. Among these TFs, Ngn3 is extremely important during development but does not participate in the CRC of mature β‐cells, while MafA and MafB are essential TSGs at later stages for β‐cell functionality. Finally, some TFs, such as Pdx1, are important in both development and in the CRC governing long‐term cell type maintenance.

The majority of enhancers, in order to influence gene expression, are located in proximity to their target gene’s promoter. Pairs of genomic loci which are nearby in 3D space can be identified using chromosome conformational capture (3C) (Dekker *et al,* 2002) or Hi‐C (Belton *et al,* 2012). More conveniently, the genome can be scanned for accessible chromatin regions. Accessibility can be assayed by DNase‐seq (Boyle *et al,* 2008) or ATAC‐seq (Buenrostro *et al,* 2015), which work by partial DNA digestion or transposases, respectively. As promoters and actively transcribed genes are also located in accessible chromatin regions, chromatin accessibility measurements need to be combined with other datasets to predict enhancers. For example, Thibodeau and colleagues were able to effectively predict enhancers from ATAC‐seq data by combining it with sequence information such as GC% and known motifs (Thibodeau *et al,* 2018).

Chromatin accessibility can also be detected indirectly by searching for associated histone or DNA modifications using ChIP‐seq (Creyghton *et al,* 2010; Rada‐Iglesias *et al,* 2011). High levels of histone H3 lysine‐27 acetylation (H3K27ac) typically mark active proximal and distal (e.g., enhancers) regulatory elements, while monomethylated H3 lysine‐4 (H3K4me1) marks primed or active enhancers in the absence or presence of H3K27ac, respectively (Shlyueva *et al,* 2014). This is a general phenomenon reported in various cell types, including human and mouse ESCs undergoing differentiation (Creyghton *et al,* 2010; Rada‐Iglesias *et al,* 2011). Tiwari *et al* (2018) integrated transcriptomic and epigenomic analyses to delineate gene regulatory programs that drive the developmental trajectory from mouse ESCs to astrocytes. By examining H3K4me1 enrichment patterns at stage‐specific H3K27ac sites during astrogliogenesis, they were able to define regulatory elements unique to each stage. Next, by inferring the most highly associated TF‐binding motifs at these elements, they unveiled drivers of the underlying differentiation trajectory. In this way, NFIA and ATF3 were identified as drivers of astrocyte differentiation from neural precursor cells, while RUNX2 promotes astrocyte maturation (Tiwari *et al,* 2018). Another histone modification, trimethylated H3 lysine‐4 (H3K4me3), is commonly used to identify promoters (Guenther *et al,* 2007). The number and range of histone modifications that can be assessed is limited by the availability of appropriate antibodies (Satterlee *et al,* 2010), which can be of variable quality (Park, 2009). Another limitation of ChIP‐seq is the requirement for cross‐linking the DNA, which can cause epitope masking and technical artifacts (Satterlee *et al,* 2010; Baranello *et al,* 2016) meaning a large amount of starting material is required for accurate results, typically 10 million cells (Park, 2009).

High levels of H3K27ac, together with high abundance of TFs, transcriptional coactivators, and chromatin remodelers binding characterize a class of regulatory elements that have been termed super‐enhancers (Hnisz *et al,* 2013; Whyte *et al,* 2013; Moorthy *et al,* 2017). These are major regulatory components of the gene expression program that shapes cell identity (Wang *et al,* 2019). Core TFs typically bind super‐enhancers of their own genes, positively regulating their own expression, as well as the super‐enhancers of many other cell type‐specific genes, thereby establishing an interconnected regulatory network (i.e., CRC) (Hnisz *et al,* 2013; Whyte *et al,* 2013). Saint‐André and colleagues have mined super‐enhancers as an unbiased approach to identify core TFs in human ESCs, creating a map of the transcriptional regulatory circuitry involved in pluripotency and other cell lineages (Saint‐André *et al,* 2016). Integrated and interactive databases of super‐enhancers for human and mouse genomes have been made available as resources (Khan & Zhang, 2016; Qian *et al,* 2019; Jiang *et al,* 2019). Super‐enhancers have been recently identified as having a unique role in transcriptional regulation in cancer (Bradner *et al,* 2017). As oncogenic events can go hand in hand with a loss of cell identity, further investigation of transcriptional dysregulation in cancer may shed light on transcriptional programs in normal cells and unveil cell type‐specific CRCs (Bradner *et al,* 2017).

Similar to transcriptomes, epigenetic signatures can be detected at single‐cell level resolution. For example, single‐cell ATAC‐seq (scATAC‐seq) can reveal cell‐specific regulatory signatures characteristic of CRCs (Fullard *et al,* 2018). Single‐nuclear ATAC‐seq of the mouse forebrain has identified cell type‐specific genomic elements, many of which are distal enhancer elements (Preissl *et al,* 2018). TFs that bind these elements are candidate master regulators of different neuronal identities (Preissl *et al,* 2018). Recent studies based on single‐nucleus methylomes have also expanded the atlas of brain cell types and identified regulatory elements that drive conserved brain cell diversity (Luo *et al,* 2017). Taken together, these studies highlight how studying chromatin modifications across different cell types can help identify candidate CRCs.

Although single‐cell epigenomics has allowed more precise cell type‐specific modifications to be detected, it faces similar challenges to those of scRNA‐seq. Assays based on single‐cell sequencing require amplification strategies and individual cell isolation which limit the analysis of cells of lower abundancy and end‐sequencing of mRNA transcripts (Clark *et al,* 2016). For example, techniques like scATAC‐seq require a “cut and paste” mechanism to examine chromatin accessibility, which not only introduces bias but also results in extensive signal loss and generation of unusable fragments (Sun *et al,* 2019; Philpott *et al,* 2020). Furthermore, mitochondrial DNA could be present in ATAC‐seq reads (Sun *et al,* 2019). Future technological advances addressing these areas will improve the ability of ATAC‐seq to capture a whole coverage of open chromatin sites and to detect TF information.

## Computational approaches to predict CRCs

Advances in high‐throughput sequencing technologies have led to the development of multiple computational algorithms designed to predict candidate core TFs and map CRCs (Fig 2C). Some approaches allow the integration of data using gene–gene, protein–protein, gene–protein, and regulatory element interactions and provide resources and insights into basic principles governing transcriptional regulatory networks (Neph *et al,* 2012; Rolland *et al,* 2014; Saint‐André *et al,* 2016; Khan & Zhang, 2016; Qian *et al,* 2019; Jiang *et al,* 2019; Moore *et al,* 2020).

Computational methods have been established to predict the minimum combination of TFs required for inducing changes in cell identity as well as improving the efficiency of reprogramming to pluripotency (Cahan *et al,* 2014; D’Alessio *et al,* 2015; Rackham *et al,* 2016; Biddy *et al,* 2018; Nicetto & Zaret, 2019; Schiebinger *et al,* 2019). Based on just transcriptomic data, a simple method to select TFs is to calculate expression specificity for each cell type against multiple cell types. This measurement provides information about transcriptional control of cell identity and candidates can then be verified by reprogramming experiments (D’Alessio *et al,* 2015). However, this prediction may include false positives due to oversimplification. Other computational algorithms have tackled this problem by inferring GRNs. These algorithms explore and model the relationships between TFs and target genes based on the expression patterns across samples. For example, ARACNe is an algorithm which identifies potential TFs and putative target genes by “mutual information”, a measure of mutual dependence in information theory (Basso *et al,* 2005). GENIE3 is a machine learning method that infers GRNs by learning the complex co‐expression relationships of TFs and candidate target genes (Huynh‐Thu *et al,* 2010). It weighs TFs by their ability to predict expression of target genes and construct a TF network with the highest weights. A common limitation to GRNs inference is the requirement for large amounts of gene‐expression data. The data complexity, partially in the form of expression variability, is key to constructing gene‐gene relationships, such as co‐expression. This variability is assumed to be representative of perturbations in cells of identical types. Experimental conditions need therefore to be carefully designed to examine such biologically relevant variability without compromising cell identity regulation. Moreover, the mechanisms of inferred networks need to be extensively validated in experiments. The recent accumulation of reliable data of multiple omics‐types provides readily available information to direct mechanistic studies.

Multi‐omics data can also be integrated during CRC inference to make supervised predictions. Ideally, this type of data is generated from a single purified population of cells. Transcriptomic data inform TF‐target gene relationships and co‐factor expression, which may indicate phenotypic specificity of the regulatory complex. TF activity on target genes can be inferred from ChIP‐seq peak profiles, especially when they overlap with active promoters (H3K4me3 peaks) and enhancers (H3K27ac peaks). Chromosome conformation capture technologies can provide a basis for cell type‐specific predictions of enhancer‐promoter interactions. Also, TF activity can be studied by looking at overlapping peaks mapped by DNase‐seq or ATAC‐seq. Some of these epigenetic data are available in databases, such as ENCODE (Dunham *et al,* 2012), Roadmap Epigenetics (Roadmap Epigenomics Consortium *et al,* 2015), Blueprint (Martens & Stunnenberg, 2013), and GeneHancer (Fishilevich *et al,* 2017), especially for stable cell lines. Furthermore, experimental protein–protein interaction information curated in databases, such as StringDB (Szklarczyk *et al,* 2018), IntAct (Orchard *et al,* 2014), and BioGrid (Oughtred *et al,* 2018), enable consideration of co‐factors and TF complexes in building a CRC. While acquiring deep multi‐omics data is not yet technologically feasible, computational algorithms have been designed to integrate data from various databases with minimum user input for CRC inference. SCENIC infers TF GRNs using GENIE3 and applies *RcisTarget* to further reduce false‐positive TF‐target gene relationships in the network by performing cis‐regulatory motif analysis (Aibar *et al,* 2017). While intended for single‐cell data, it is applicable to any transcriptomic datasets with sufficient size and complexity. The recently developed computational method *M*ulti‐*o*mics *n*etwork *i*nference (Moni) integrates TF ChIP‐seq data, protein–protein interactions, enhancer–promoter interactions, and reference RNA‐seq data from databases, along with individual user input of cell type‐specific datasets to identify TFs and co‐factors, and eventually reconstructs enhancer‐promoter GRNs (Jung & del Sol, 2020). Although this approach is limited by the multi‐omics data available for each cell type, the construction of precise CRCs will become more feasible as “omics” technologies mature and become more affordable. Advances in computational methods will need to be pursued in parallel to address challenges associated with data integration—to link data from heterogeneous sources and different measurement types with increased complexity and perform correction of batch effects.

The availability of single‐cell data has inspired novel approaches to integrate data. Schiebinger *et al* (2019) applied the mathematical concept of optimal transport, which efficiently computes a distance between distributions, to scRNA‐seq profiles of mouse embryonic fibroblasts undergoing reprogramming to pluripotent stem cells. TFs predictive of various fates were inferred and then experimentally tested. Among others, the homeobox *Obox6* was found to correlate strongly with the pluripotent cell state and when combined with OCT4, KLF4, SOX2, and MYC factors, it enhanced reprogramming efficiency (Schiebinger *et al,* 2019). However, as previously mentioned, while single‐cell transcriptomics allow identification of new cell types and states, an immediate challenge is to establish and validate consensus assignment of cell types and states, and to standardize experimental procedures to generate comparable results. Future techniques that enable collection of multiple data types from single cells or a highly homogenous population will (1) enhance the granularity and precision of CRC inference methods, (2) help to gain deeper insights into the complexity of CRCs, such as the hierarchy and molecular mechanisms of regulation, and (3) enable characterization of variability of CRCs across time, cell states and types.

In developmental systems, the concept of cell state refers to cell fate transition along a particular developmental trajectory (Kester & van Oudenaarden, 2018). With time included as a variable, more advanced computational methods are needed that can integrate data across a time scale. An example is dynGENIE3, which infers dynamical GRN models from time series expression data (Huynh‐Thu & Geurts, 2018). These methods are particularly useful for studying changing GRN along the developmental trajectory. Furthermore, cross‐sectional single‐cell data that captures continuous developmental stages can be reconstructed into pseudo time‐series. More than 50 bioinformatics tools have been developed for pseudotime analyses in scRNA‐seq data (Saelens *et al,* 2019; Tritschler *et al,* 2019). Beyond embryonic development, single‐cell pseudotime approaches provide powerful means for identification of differentiation trajectories in adult stem cell compartments as well as in disease (Tritschler *et al,* 2019). Since pseudotime analysis orders cells according to their overall transcriptomic similarity (Trapnell *et al,* 2014), it should be interpreted as a relative measure of cellular differentiation state, or maturity, rather than one on an absolute time‐scale. Once such a trajectory is inferred, the transcriptional cell states at which fate decisions are made and the TFs driving these decisions can potentially be identified. For example, pseudotime series can be used to calibrate models such as non‐linear ordinary differential equation models (Ocone *et al,* 2015) that capture CRC dynamics. It should be noted that pseudotime approaches cannot replace traditional cellular lineage tracing techniques (Kester & van Oudenaarden, 2018) but the two are complementary and should be used to validate each other.

## Assigning functional relevance to CRCs

Transcriptomics, epigenetics, and computational approaches can be used to predict CRC components and architecture. However, functional experiments, such as genetic perturbation and reprogramming studies, provide validation of these predictions. An important challenge is to match transcriptional and functional profiles of a cell population. In some cases, cells with apparently identical CRCs exhibit disparate functions. A compelling example of this is the existence of dynamic, interchangeable states between pancreatic β‐cells, which are likely to be controlled at multiple levels and influenced by the pancreatic islet microenvironment (Dominguez‐Gutierrez *et al,* 2019); here, the existence of multiple combinations of states may correlate with varying levels of insulin secretion. Diverse calcium responses to glucose stimulation are also found among β‐cells, and this has recently been shown to further fluctuate when the cells are detached from their host islets (Scarl *et al,* 2019). Functional differences of this kind have been reconciled in the context of neuronal subtypes possessing the same CRC, where the activity of terminal selectors was demonstrated to vary in the presence of repressor proteins confined to a specific cell subtype, thereby curbing the expression of the terminal gene battery (Hobert, 2016). Consequently, there exist limitations when considering cell identity exclusively from a molecular or functional perspective and the CRC should not be considered in isolation.

The concept of CRC not only provides a more comprehensive way of defining cell identities but might also have direct implications in regenerative medicine. For example, the knowledge of core TFs and CRCs underlying a desired cell identity may have a direct impact in lineage reprogramming and advance its clinical translation. Indeed, direct lineage reprogramming represents a strategy for generating desired functional cells that can be used in cell therapies (Heinrich *et al,* 2015), as the idea underlying successful lineage reprogramming is based on the knowledge of transcriptional networks governing cellular identity (Graf & Enver, 2009; Heinrich *et al,* 2015). A lot of progress has been made in this field, since it is now possible to obtain an array of different cellular types from distinct mature populations (Zhou *et al,* 2008; Vierbuchen *et al,* 2010; Heinrich *et al,* 2015; Xu *et al,* 2015). Direct lineage reprogramming unfolds developmental programs and argues for the engagement of hierarchical developmental CRCs, providing another strategy for discovering CRC factors.

## Concluding remarks

We propose that the CRCs provide a comprehensive and uniform framework for defining the identity of a cell. The significant increase in our understanding of gene expression, particularly from single‐cell datasets, underscores the need to unify and integrate new information with prior knowledge. At the same time, these new insights have reopened the definitions of a cell's identity. While resources such as the HCA has given us access to information about each cell of the human body, additional functional studies are required to build a complete map of CRCs. In particular, the knowledge inferred from all the available datasets has to be tested in human models using high‐throughput approaches, such as CRISPR‐based screening platforms in human pluripotent stem cells. Large‐scale observational studies offer another way to assess the relevance of suspected CRC components in a human setting. For example, the Human Knockout Project (https://www.broadinstitute.org/cardiovascular/human‐knockout‐project) studies loss‐of‐function phenotypic consequences in naturally occurring human genetic variants. Besides assessing the functional relevance of TFs, a fundamental challenge is to combine the transcriptome and epigenomic characterization of individual cell types with concurrent CRISPR‐based genome and enhancer‐targeting editing approaches. Only such a systems approach will elucidate if a CRC is self‐sustaining and drives the expression of genes, which maintain unique cellular traits. Finally, future efforts will be directed to integrate core TFs with cell behavior and function into a more comprehensive concept of cell regulatory networks.

## Conflict of interest

F.M.W. is currently on secondment as executive chair of the UK Medical Research Council. The other authors declare that they have no conflict of interest.
